# What factors should we modify to promote high functioning and prevent functional decline in people with schizophrenia?

**DOI:** 10.3389/fpsyt.2023.1181758

**Published:** 2023-06-02

**Authors:** Clara Martínez-Cao, Ainoa García-Fernández, Leticia González-Blanco, Paula Zurrón-Madera, Pilar A. Sáiz, María Paz García-Portilla, Julio Bobes

**Affiliations:** ^1^Department of Psychiatry, Universidad de Oviedo, Oviedo, Spain; ^2^Instituto de Investigación Sanitaria del Principado de Asturias (ISPA), Oviedo, Spain; ^3^Instituto Universitario de Neurociencias del Principado de Asturias (INEUROPA), Oviedo, Spain; ^4^Servicio de Salud del Principado de Asturias (SESPA) Oviedo, Oviedo, Spain; ^5^CIBER de Salud Mental, Instituto de Salud Carlos III, Madrid, Spain

**Keywords:** real-world functioning, protective factors, risk, prevention, schizophrenia

## Abstract

**Background:**

Since research in schizophrenia mainly focuses on deficits and risk factors, we need studies searching for high-functioning protective factors. Thus, our objective was to identify protective (PFs) and risk factors (RFs) separately associated with high (HF) and low functioning (LF) in patients with schizophrenia.

**Methods:**

We collected information (sociodemographic, clinical, psychopathological, cognitive, and functional) from 212 outpatients with schizophrenia. Patients were classified according to their functional level (PSP) as HF (PSP > 70, *n* = 30) and LF (PSP ≤ 50, *n* = 95). Statistical analysis consisted of Chi-square test, Student’s *t*-test, and logistic regression.

**Results:**

HF model: variance explained: 38.4–68.8%; PF: years of education (OR = 1.227). RFs: receiving a mental disability benefit (OR = 0.062) and scores on positive (OR = 0.719), negative-expression (OR = 0.711), and negative-experiential symptoms (OR = 0.822), and verbal learning (OR = 0.866). LF model: variance explained: 42.0–56.2%; PF: none; RFs: not working (OR = 6.900), number of antipsychotics (OR = 1.910), and scores on depressive (OR = 1.212) and negative-experiential symptoms (OR = 1.167).

**Conclusion:**

We identified specific protective and risk factors for high and low functioning in patients with schizophrenia and confirmed that high functioning factors are not necessarily the opposite of those associated with low functioning. Only negative experiential symptoms are a shared and inverse factor for high and low functioning. Mental health teams must be aware of protective and risk factors and try to enhance or reduce them, respectively, to help their patients improve or maintain their level of functioning.

## 1. Introduction

Schizophrenia is one of the leading causes of severe disability in adults (1) since it affects patients’ ability to live independently, be socially active, and work or study. Therefore, there has been growing interest in identifying clinical factors associated with functioning in these patients (2). Having negative symptoms has been suggested as one of the most important predictors of low real-world functioning (3–9). Furthermore, some authors suggest specific relationship patterns. While experiential symptoms (avolition, anhedonia, and asociality) negatively impact work, global functioning, and interpersonal relationships (10–13), expressive symptoms (affective flattening and alogia) mainly impact social functioning (14–16).

Different cognitive dimensions have also been associated with functioning (2, 17–21). For example, in the meta-analysis by Cowman et al. (2), general cognitive ability and social cognition were the dimensions most strongly associated with psychosocial functioning. On the contrary, attention deficits seem to negatively and significantly impact daily functioning (18). Research also reflects an association between impaired real-world functioning with communicative-pragmatic abilities (22), positive (6, 7), depressive (19, 23), extrapyramidal (23), and autistic symptoms (24).

Concerning the impact of disease duration on real-world functioning, the results reported so far are conflicting. For example, Costa et al. (25) did not find significant differences in functioning based on years of illness. On the contrary, in a meta-analysis conducted by Winter et al. (26), better social functioning was associated with fewer negative symptoms, better quality of life, and better occupational functioning in the early years of the disorder. Likewise, Liemburg et al. (27) found that avolition could mediate the association between cognition and occupational performance in first-episode patients, while expressive deficits mediated this association in patients with greater length of illness.

With the above in mind and given that research in psychiatry has traditionally focused on deficits and risk factors for mental disorders (28, 29), Santesteban-Echarri et al. (30) pointed out that there is a need for studies to search for factors that protect good functioning. Furthermore, to our knowledge, most studies try to identify factors associated with patients’ level of functioning instead of identifying those specific factors related to having a high or low level of functioning. However, increased knowledge about the factors associated with high and low levels of functioning will allow us to develop better personalized intervention programs, thus improving patient prognosis.

Therefore, the present study aims to identify the protective and risk factors separately associated with high and low functioning in patients with schizophrenia. Furthermore, since factors associated with poor functioning are better established, one of our main objectives is identifying the factors related to high functioning. We hypothesize that they need not necessarily be the opposite of those associated with low functioning.

## 2. Methods

### 2.1. Study design

This is a naturalistic, cross-sectional study conducted in Oviedo, Spain in accordance with the ethical principles of the Declaration of Helsinki and Good Clinical Practice guidelines. It received the approval of the Clinical Research Ethics Committee of Hospital Universitario Central de Asturias in Oviedo, Spain (Ref. 36/2012, 25/2014). All participants gave their written informed consent before enrolment.

### 2.2. Study participants

The number of participants recruited was 212 patients with stable schizophrenia. Stability was defined as those patients who were clinically stable and had not required any change in their current pharmacological treatment during the last 3 months. To increase the external validity of our results and, therefore, their generalization to daily clinical practice, the exclusion and inclusion criteria to participate in the study were minimal. Inclusion criteria were (1) diagnosis of schizophrenia according to ICD-10 (International Classification of Diseases 10th Revision) criteria, (2) over 17 years of age, (3) receiving outpatient treatment, and (4) written informed consent to participate in the study. Exclusion criteria were designed to be minimal, and only those with an intellectual developmental disorder or an acquired brain injury, or who refused to participate in the study were excluded.

### 2.3. Measures

Participants were assessed by trained psychologists. The evaluation included an *ad hoc* questionnaire to collect sociodemographic and clinical data. Spanish versions of the following instruments: (1) Psychopathology: Positive and Negative Syndrome Scale (PANSS) (31), Clinical Assessment Interview of Negative Symptoms (CAINS) (32), the Calgary Depression Scale for Schizophrenia (CDSS) (33), and the Clinical Global Impression-Schizophrenia scale (CGI-SCH) (34) were also used to assess the severity of the illness. The following were also used: (2) Cognition: Measurement and Treatment Research to Improve Cognition in Schizophrenia (MATRICS) (35), and (3) Functioning: Personal and Social Performance Scale (PSP) (36).

The CAINS is a new instrument designed to assess the negative syndrome of schizophrenia focusing on the subjective experience of patients. Therefore, it was necessary to develop a semi-structured interview lasting approximately 30 min to apply this scale. It has two subscales: motivation and pleasure (MAP), which evaluates the severity of avolition and anhedonia in occupational, recreational, and social activities (9 items), and expression (EXP), which measures the severity of alogia and blunted affect (4 items). Items are scored on a 5-point scale (0–4), with higher scores reflecting greater impairment. It provides scores for each subscale: for MAP, the total score ranges from 0 to 36, and for the EXP subscale, the total score ranges between 0 and 16. It also provides a total score obtained by combining the two subscale scores, with higher scores reflecting greater severity (0–52).

The PSP is one of the scales most used to assess real-world functioning by evaluating the following four dimensions: self-care, socially useful activities including work and study, personal and social relationships, and disturbing and aggressive behaviors. It provides a total score ranging from 0 (worst possible level of functioning) to 100 (optimal functioning). To address our objective, the cut-off points proposed in this scale were used to classify patients (37). Therefore, those with scores greater than 71, reflecting mild or no functional difficulties, were included in the high-functioning group. Similarly, the low-functioning group included patients with scores below 50, reflecting severe functional impairment.

### 2.4. Statistical analyses

Data were analyzed using IBM SPSS Version 24.0 (IBM Corp., Armonk, NY). The two-tailed level of significance used was <0.050. Data are presented as mean (standard deviation [SD]) for continuous variables and as frequencies and percentages for categorical variables. To achieve the aim proposed in our study, patients were classified into four groups according to the level of functioning evaluated by the PSP. Firstly, we created a group called High Functioning (HF) that included patients with a PSP score of 71 to 100 (*n* = 30); this group was compared with the rest of the sample (RS_H_, PSP score of ≤ 70, *n* = 182). Secondly, we classified patients with a PSP score of 0 to 50 (*n* = 95) in a group called Low Functioning (LF) to compare them with the rest of the sample (RS_L_, PSP score ≥ 51, *n* = 117). Finally, we conducted two logistic regressions (stepwise forward method) to determine the independent factors associated with LF and HF. *A priori*, we did not include total scale scores or any redundant measure to avoid collinearity. In addition, we obtained the variance inflation factor (VIF) to diagnose collinearity between the independent variables included in the regression análisis (no collinearity = VIF values below 5).

## 3. Results

The mean age of the total sample was 40.30 (13.50), 63.70% were males, and 59.50% had a secondary education. Most patients had never been married (74.10%), 14.60% were working, and more than a third were receiving disability benefits due to schizophrenia (37.70%).

The mean length of the disease was 11.98 (12.02) years. Most had at least one hospitalization (70.80%), and 16.00% had a history of attempted suicide. Concerning real-world functioning scores, the mean PSP total score was 53.54 (17.67). More detailed information is available under request.

### 3.1. Differences among the groups

Of the total sample, 30 (14.15%) patients belonged to the HF, and 95 (44.81%) to the LF group. The patients with HF were significantly younger, and in a higher proportion woman than the RS_H_. In addition, they had a higher educational level and more years of education, shorter duration of illness, fewer hospitalizations, and less use of antipsychotics, benzodiazepines, and antidepressants (see Table 1). Regarding psychometric scores, HF patients had significantly lower negative, positive, and depressive scores. This group scored higher in all dimensions of cognition, but we only found statistically significant differences in working memory, verbal and visual learning, and social cognition (Table 2).

**Table 1:** Sociodemographic and clinical characteristics based on the level of functioning.

	HF (*n* = 30)		RS_H_ (*n* = 182)		Statistical test, *p*	LF (*n* = 95)		RS_L_ (*n* = 117)		Statistical test, *p*
	*n*	%	*n*	%		*n*	%	*n*	%	
Age [mean (sd)]	33.97	9.85	41.34	13.24	3.596^a^, <0.001	42.51	13.15	38.50	12.78	−2.240^a^, 0.026
Sex: males	14	46.66	121	66.48	4.373^b^, 0.037	63	66.31	72	61.53	0.517^b^, 0.472
Marital status					0.124^b^, 0.725					3.165^b^, 0.075
Never married	23	76.66	134	73.62		76	80.00	81	69.23	
Married^*^	7	23.33	48	26.37		19	20.00	36	30.76	
Education level					9.095^b^, 0.011					5.177^b^, 0.075
Primary school	2	6.66	44	24.17		21	22.10	25	22.22	
Secondary school	17	56.66	108	59.34		62	65.26	63	53.84	
Post-secondary school	11	36.66	30	16.48		12	12.63	29	24.78	
Years of education [mean (sd)]	17.13	4.64	13.43	4.28	−4.327^a^, <0.001	13.35	3.80	14.45	4.98	1.831^a^, 0.069
Work status					10.829^b^, 0.004					28.071^b^, <0.001
Working	8	26.66	23	12.63		3	3.15	28	23.93	
Not working^#^	14	46.66	138	75.82		85	89.47	67	57.26	
Homemaker or student	8	26.66	21	11.53		7	7.36	22	18.80	
Mental disability benefit (yes)	1	3.33	79	43.40	17.602^b^, <0.001	54	56.84	26	22.22	26.744^b^, <0.001
Length of illness [mean (sd)]	6.63	7.49	12.86	12.41	3.776^a^, <0.001	13.77	12.72	10.52	11.26	−1.969^a^, 0.050
Hospitalizations (yes)	22	73.33	128	70.32	0.112^b^, 0.738	67	70.52	83	70.94	0.004^b^, 0.947
No. of hospitalizations [mean (sd)]	1.13	1.00	1.70	1.99	2.390^a^, 0.019	1.68	1.91	1.56	1.89	−0.457^a^, 0.648
Age at first hospitalization [mean (sd)]	26.76	5.04	29.49	9.56	1.966^a^, 0.055	30.37	10.17	28.07	8.05	−1.541^a^, 0.126
Suicide attempts (yes)	4	13.33	30	16.48	0.190^b^, 0.663	24	25.26	10	8.54	10.880^b^, <0.001
No. of suicide attempts [mean (sd)]	0.17	0.46	0.29	0.91	0.729^a^, 0.467	0.41	0.97	0.16	0.75	−2.038^a^, 0.043
Number of antipsychotics [mean (sd)]	1.07	0.64	1.40	0.73	2.349^a^, 0.020	1.58	0.76	1.17	0.64	−4.131^a^, <0.001
Antidepressants (yes)	2	6.66	48	26.37	5.555^b^, 0.018	30	31.57	20	17.09	6.104^b^, 0.013
Benzodiazepines (yes)	7	23.33	96	52.74	8.920^b^, 0.003	63	66.31	40	34.18	21.664^b^, <0.001

HF, high functioning; RS_H_, rest of the sample for the HF group; LF, low functioning; RS_L_, rest of the sample for the LF group; sd, standard deviation. ^*^Married includes married, living as married, widowed, and divorced; ^#^Not working includes due to permanent or temporary physical disability, retirement, and unemployment.

a Student *t*-test.

b Chi-square test.

**Table 2:** Psychometric, cognitive, and functioning scores of the four functioning groups.

	HF (*n* = 30)		RS_H_ (*n* = 182)		Statistical test, *p*	LF (*n* = 95)		RS_L_ (*n* = 117)		Statistical test, *p*
	Mean	sd	Mean	sd		Mean	sd	Mean	sd	
Psychometric scores										
PANSS-Positive	10.00	2.72	13.37	5.25	5.334^a^, <0.001	14.55	5.06	11.56	4.75	−4.423^a^, <0.001
PANSS-Negative	11.67	3.58	19.29	5.12	7.831^a^, <0.001	21.14	4.84	15.83	5.02	−7.770^a^, <0.001
CAINS-Total	11.27	7.59	30.49	10.75	12.019^a^, <0.001	35.22	8.61	21.73	11.59	−9.423^a^, <0.001
CAINS-MAP	9.07	5.89	22.75	7.86	9.115^a^, <0.001	26.43	6.11	16.26	8.34	−9.918^a^, <0.001
CAINS-EXP	2.20	2.88	7.74	4.30	9.005^a^, <0.001	8.79	4.07	5.47	4.40	−5.641^a^, <0.001
CDSS	1.10	1.58	3.51	4.21	5.664^a^, <0.001	4.92	4.92	1.75	2.33	−5.756^a^, <0.001
CGI-Global	2.93	0.58	4.34	0.81	11.909^a^, <0.001	4.80	0.66	3.68	0.81	−10.994^a^, <0.001
Cognition scores (MATRICS)										
Speed of Processing	36.86	11.15	31.99	15.50	−1.651^a^, 0.100	29.14	13.22	35.55	15.84	3.152^a^, 0.002
Attention	35.46	11.31	33.83	11.19	−0.741^a^, 0.460	32.25	10.23	35.52	11.75	2.136^a^, 0.034
Working Memory	44.80	10.71	37.69	13.02	−2.832^a^, 0.005	35.95	12.56	40.93	12.85	2.830^a^, 0.005
Verbal Learning	42.26	11.13	38.21	10.08	−2.009^a^, 0.046	36.08	8.47	40.98	11.14	3.632^a^, <0.001
Visual Learning	44.33	14.40	35.16	13.21	−3.476^a^, <0.001	32.82	11.62	39.41	14.63	3.649^a^, <0.001
Reasoning	38.43	8.31	36.97	9.64	−0.783^a^, 0.435	35.45	9.49	38.58	9.24	2.421^a^, 0.016
Social Cognition	48.69	18.68	40.27	15.69	−2.648^a^, 0.009	38.76	15.03	43.66	17.12	2.184^a^, 0.030
Functioning scores										
PSP-Total	81.67	6.75	48.90	14.28	−20.161^a^, <0.001	37.28	8.83	66.74	10.62	21.621^a^, <0.001
PSP-Self-care	0.37	0.49	1.42	1.03	8.912^a^, <0.001	1.84	1.11	0.80	0.69	−7.915^a^, <0.001
PSP-Socially useful activities, including work and study	0.73	0.58	2.75	1.07	15.174^a^, <0.001	3.44	0.64	1.68	1.02	−15.275^a^, <0.001
PSP-Personal and social relationships	0.70	0.53	2.66	0.93	16.383^a^, <0.001	3.22	0.63	1.70	0.94	−13.879^a^, <0.001
PSP-Disturbing and aggressive behaviors	0	0	0.20	0.52	5.135^a^, <0.001	0.29	0.63	0.07	0.28	−3.226^a^, 0.002

HF, high functioning; RS_H_, rest of the sample for the HF group; LF, low functioning; RS_L_, rest of the sample for the LF group; sd, standard deviation; PANSS, positive and negative syndrome scale; CAINS, clinical assessment interview for negative symptoms; CAINS-EXP, expression subscale; CAINS-MAP, motivation and pleasure subscale; CDSS, Calgary depression scale for Schizophrenia; CGI, clinical global impression-severity; MATRICS, measurement and treatment research to improve cognition in Schizophrenia; PSP, personal and social performance.

a Student *t*-test.

On the other hand, patients with LF were significantly older than the RS_L_. We also found statistically significant differences in employment status and perception of mental disability benefits. In addition, these patients had a higher percentage of suicide attempts and were prescribed more antipsychotic, benzodiazepine, and antidepressant drugs (Table 1). Concerning psychometrics, LF patients had significantly higher negative, positive, and depressive scores than the other group. Finally, this group obtained statistically significant lower scores in all cognitive dimensions than the RS_L_ (see Table 2).

### 3.2. Models for each level of functioning groups

#### 3.2.1. Protective and risk factors for high functioning

All factors significantly related to HF in the univariate analysis were included in the logistic regression analysis because the VIF values obtained for these variables ranged between 1.1 and 3.4. Our model explained between 38.4 and 68.8% of the variance (Cox & Snell R^2^ and Nagelkerke R^2^, respectively). The factors retained in the model are shown in Table 3. Years of education contributed to belonging to the HF group (OR = 1.227). On the contrary, receiving a mental disability benefit (OR = 0.062) and scores on positive symptoms (OR = 0.719), CAINS-EXP (OR = 0.711), CAINS-MAP (OR = 0.822), and verbal learning (OR = 0.866) reduced the probability of being in the HF group (see Table 3).

**Table 3:** Logistic regression models for high (HF) and low functioning (LF).

	Β	SE	Wald	df	*p*	OR	95% CI
High Functioning							
Intersection	9.176	2.873	10.203	1	0.001		
Years of education	0.204	0.084	5.954	1	0.015	1.227	1.041–1.446
Mental disability benefit (yes)	−2.786	1.281	4.729	1	0.030	0.062	0.005–0.760
PANSS-Positive	−0.329	0.106	9.727	1	0.002	0.719	0.585–0.885
CAINS-MAP	−0.196	0.064	9.384	1	0.002	0.822	0.725–0.932
CAINS-EXP	−0.342	0.115	8.836	1	0.003	0.711	0.567–0.890
Verbal Learning	−0.143	0.049	8.578	1	0.003	0.866	0.787–0.954
Correct predictions	92.90						
Low Functioning							
Intersection	−6.645	1.067	38.820	1	<0.001		
Work status, reference: working							
Not working	1.932	0.788	6.005	1	0.014	6.900	1.472–32.346
Homemaker or student	0.626	0.927	0.456	1	0.500	1.870	0.304–11.516
Number of antipsychotics	0.647	0.290	4.983	2	0.026	1.910	1.082–3.370
CAINS-MAP	0.155	0.028	29.805	1	<0.001	1.167	1.104–1.234
CDSS	0.192	0.061	10.102	1	0.001	1.212	1.077–1.365
Correct predictions	78.30						

PANSS, positive and negative syndrome scale; CAINS, clinical assessment interview for negative symptoms; CAINS-EXP, expression subscale; CAINS-MAP, motivation and pleasure subscale; CDSS, Calgary depression scale for Schizophrenia; SE, standard error; df, degrees of freedom; OR, odds ratio; CI, confidence interval.

#### 3.2.2. Protective and risk factors for low functioning

We developed a second logistic regression model to identify factors associated with LF. Before doing so, we verified that the variables introduced into the regression were not collinear. The variables significantly related to LF in the univariate analysis had VIF values between 1.1 and 2.5; therefore, all of them were included in the logistic regression. Our model explained between 42.0 and 56.2% of the variance (Cox & Snell R^2^ and Nagelkerke R^2^, respectively). The model retained the following risk factors: not working (OR = 6.900), the number of antipsychotics (OR = 1.910), scores on depressive symptoms (OR = 1.212), and CAINS-MAP (OR = 1.167) (see Table 3).

## 4. Discussion

To our knowledge, this is one of the few studies that tries to separately identify protective and risk factors for a high and low level of functioning in patients with schizophrenia. As hypothesized, we have identified specific factors associated with high functioning that are not the opposite of those related to low functioning. Only the motivation and pleasure dimension of the negative symptomatology construct is a common risk factor for both levels of functioning; the higher its severity, the lower the probability of belonging to the HF group and the higher the likelihood of belonging to the LF group.

### 4.1. Protective and risk factors for high functioning

Achieving a high educational level would be a sociodemographic variable to consider for improving patient functioning, since it contributes positively to belonging to the HF group. Previous studies support our results; higher education is associated with better cognitive and functional performance (24, 38). One possible explanation for these results is that education is one of the factors that contribute to cognitive reserve (CR). This theoretical concept can explain the differences observed in late-life cognitive impairment (39). Research interest in CR has increased in recent years, and it may be an essential concept for better understanding the different functional outcomes of patients with schizophrenia (40).

We also showed that receiving a benefit for mental disability decreases the probability of belonging to the HF group. Although this finding might seem logical based on the reasoning that those who receive this social benefit are the most severely ill patients, other factors could be influencing it in some instances. We want to point out that this factor was retained in the equation along with positive and negative symptoms; thus, it has its specific weight in decreasing the probability of belonging to the HF group. Receiving an economic benefit from the government can prevent people from trying to improve their occupational skills and obtain a protected or regular job, either because they already have their basic needs covered or because they fear their future as workers and the possibility of losing this benefit. Social regulations that are more flexible and adjusted to these situations can help to overcome these barriers and promote the recovery of patients with schizophrenia.

The presence of positive and negative symptoms decreased the probability of being in the HF group. These results agree with previous research; negative and positive symptoms were associated with worse functional outcomes (3, 4, 6, 30, 41–44). The scientific literature also shows that positive symptoms have less impact on global functioning than negative symptoms (45), which is consistent with our findings. However, it should be noted that CAINS-MAP was a factor that emerged in both functioning groups (HF and LF). On the contrary, CAINS-EXP was identified as a significant predictor in the HF group only; the greater the intensity of expressive negative symptoms, the lower the probability to belong to the HF group. Therefore, this finding highlights the need for early intervention in this area to prevent functional deterioration. Helpful specific strategies would include avoiding antipsychotic-induced parkinsonism, antipsychotic/benzodiazepine-induced sedation, and depression.

In our study, within the cognitive dimensions, verbal learning was the one that emerged as a significant risk factor for the HF group. Previous research reflects the strong association between neurocognition and social cognition with functioning (3, 17, 18, 42, 46–50). However, contrary to our expectations, higher verbal learning scores were associated with a lower probability of belonging to the HF group. These results should be interpreted bearing in mind that it is difficult to assess the cognitive functions that affect patients’ lives using a neurocognitive test alone (18). Therefore, it should be helpful to also assess patient functional capacity, which mediates the relationship between global cognitive ability and real-world functioning (6, 42). Nevertheless, we would point out that, consistent with our results, Yang et al., (4) also found an inverse association, although it did not reach statistical significance.

### 4.2. Protective and risk factors for low functioning

Surprisingly, we did not identify any protective factor for LF.

It is essential to point out that not working emerged as one of the most critical risk factors of the LF group. Our results corroborate the relationship between functional impairment and employment (51, 52). Patients must deal with symptoms, stigma, and discrimination, a significant barrier in the job search process. In fact, in a 10 year follow-up study, 48% of the sample never obtained employment during the follow-up period (53). Thus, the occupational and social dimensions are essential objectives for patient recovery. For this reason, sheltered employment programs along with counseling and job search programs are crucial. In addition, since sustained work is associated with a better prognosis (54), as work provides financial remuneration and improves overall community participation, social activities, and quality of life (55). Work support programs are also worthwhile.

Number of antipsychotics was identified as the second most important risk factor for LF. Although antipsychotics have not been approved to improve functioning in patients with schizophrenia, they might mediate the association between psychopathology and real-world functioning (56). Previous studies reflect associations between symptomatic response to antipsychotics and better functional outcomes (56–58). Furthermore, an increased number of antipsychotics could indirectly measure illness severity (59), since patients with more severe illness may need higher doses of antipsychotics or more complex antipsychotic treatment. On the other hand, a review by Baigianti et al. (60) found that less antipsychotic medication predicted better results in cognitive remediation therapy. In this sense, antipsychotic treatment could deteriorate cognitive functions (59, 61, 62) that correlate with functioning outcomes (2, 17–21).

We also showed that depressive symptoms were significantly linked to a higher probability of belonging to the LF group. Previous research confirms these results (19, 23, 63), and further support was provided by two recent network analyses by Galderisi et al. (64) and Dal Santo et al. (65) where depression emerged as a significant node associated with functioning. Specifically, Galderisi et al. (64) found that avolition mediated this association. However, in our study, avolition and anhedonia emerged as the fourth most crucial risk factor for belonging to the LF group, with a lower weight than depressive symptoms. In this sense, Rocca et al. (63) also found that depressive symptoms decreased the probability of having good functioning and even had a greater weight than negative symptoms. Therefore, these results make us reflect on the importance of depressive symptoms in functioning. Traditionally negative symptoms have been suggested as one of the most critical predictors (3–16, 66); however, when depressive symptoms are included, it seems that they could play an even more important role in predicting real-world functioning (63).

Finally, we would like to provide some reflections on the impact of duration of illness on functioning in these patients. Although in the univariate analysis, the HF group had a significantly shorter duration of the disorder than the LF group, when we included this variable in the logistic regression together with the rest of the variables, it was not retained in the model. Our result contradicts several studies that found an association between longer disease duration and poorer functioning (56, 67–72). This discrepancy fuels the debate about whether longer illness duration is invariably and unequivocally associated with poorer functioning in people with schizophrenia. Accepting this assumption as accurate would lead to therapeutic and scientific nihilism since disease duration is currently an unmodifiable factor. Consequently, on the one hand, there would be no possibility of functional recovery or even improvement despite new pharmacological and psychosocial interventions developed to date (73–75). On the other, this would be a false statement for those patients whose functioning has been severely impaired since the first years of diagnosis (11, 62, 76, 77). In these cases, there may be a risk of not making maximum therapeutic efforts from the first months of the disease to achieve functional recovery due to the false belief that the initial phases are associated, at best, with minimal functional deterioration.

One of the main limitations of this study was its cross-sectional design. Although from a statistical point of view, the use of “risk” and “protective factors” is more appropriate for longitudinal studies, we have used them for better understanding and translation into clinical applicability. In the case of the HF group, protective factors refer to those variables increasing the likelihood of belonging to this group and risk factors to those that decrease this probability. The opposite applies to the LF group. Another significant limitation is the small sample size of HF group, which may affect generalization of the results. This limitation is determined by the strict cut-off point that we use to define HF (PSP score > 70).

Among the strengths of the study, we want to highlight the use of the CAINS to assess the negative symptoms of schizophrenia, which allowed us to avoid an overlap with functioning – the main outcome of this study – that characterizes the most traditionally used scales (e.g., PANSS). In addition, since the inclusion and exclusion criteria were not restrictive, both the representativeness of our sample and the generalizability of our results to patients in real practice is high.

### 4.3. Conclusion

Our study, unlike others, identified specific protective and risk factors associated with high and low functioning in patients with schizophrenia. It also confirmed our hypothesis that factors associated with high functioning are not necessarily the opposite of those associated with low functioning.

Only negative experiential symptoms are a shared and inverse factor for high and low functioning. In addition, we found only one specific factor associated with a high functioning level: higher education. On the contrary, we identified three specific factors related to low functioning levels: not working, having been prescribed a higher number of antipsychotics, and the presence of depressive symptoms.

From a translational point of view, mental health teams must be aware of these protective and risk factors and try to use them to help their patients improve or maintain their functioning. In this regard, careful management of antipsychotics is a crucial action that can be easily and immediately translated into daily clinical practice. Likewise, educational and job-related programs and more flexible policies on compatibility between receiving a pension and working would be of great value.

Finally, awareness on the part of professionals of the non-direct relationship between disease duration and functioning will contribute to increasing therapeutic efforts on this dimension with the hope of modifying the disability associated with this disease.

## Data availability statement

The raw data supporting the conclusions of this article will be made available by the authors, without undue reservation.

## Ethics statement

The studies involving human participants were reviewed and approved by Clinical Research Ethics Committee of Hospital Universitario Central de Asturias in Oviedo, Spain (Ref. 36/2012, Ref. 25/2014). The patients/participants provided their written informed consent to participate in this study.

## Author contributions

MG-P, PS, and JB designed the study. CM-C and MG-P performed statistical analyses and wrote the first draft of the manuscript. CM-C, MG-P, AG-F, LG-B, PZ-M, PS, and JB contributed to the collection and assessment of the sample. All authors contributed to the article and approved the submitted version.

## Funding

This work was partly supported by the Spanish Ministry of Science and Innovation, Instituto de Salud Carlos III (Ref. PI16/01761), Government of the Principality of Asturias (PCTI-2021-2023 IDI/2022/111), CIBER – Consorcio Centro de Investigación Biomédica en Red - (CB/07/09/0020), Instituto de Salud Carlos III, Ministerio de Ciencia e Innovación and Unión Europea - European Regional Development Fund. CM-C also thanks the Ministry of Science, Innovation, and Universities for its FPU grant (FPU19/01231), and AG-F thanks Instituto de Salud Carlos III for its PFIS grant (FI20/00318).

## Conflict of interest

The authors declare that the research was conducted in the absence of any commercial or financial relationships that could be construed as a potential conflict of interest.

## Publisher’s note

All claims expressed in this article are solely those of the authors and do not necessarily represent those of their affiliated organizations, or those of the publisher, the editors and the reviewers. Any product that may be evaluated in this article, or claim that may be made by its manufacturer, is not guaranteed or endorsed by the publisher.
